# Association between Insulin-Like Growth Factor-1 and Relative Skeletal Maturation: A Retrospective Cohort Study of Short Children and Adolescents

**DOI:** 10.1155/2020/8052143

**Published:** 2020-08-13

**Authors:** Qianqian Zhao, Mei Zhang, Yuntian Chu, Baolan Ji, Hui Pan, Hailing Sun, Bo Ban

**Affiliations:** ^1^Department of Endocrinology, Affiliated Hospital of Jining Medical University, Jining Medical University, 89 Guhuai Road, Jining, Shandong P.R. 272029, China; ^2^Chinese Research Center for Behavior Medicine in Growth and Development, 89 Guhuai Road, Jining, Shandong P.R. 272029, China; ^3^School of Health Management and Medicine, Tongji Medical College, Huazhong University of Science and Technology, Wuhan, Hubei P.R. 430030, China; ^4^Key Laboratory of Endocrinology of National Health and Family Planning Commission, Department of Endocrinology, Peking Union Medical College Hospital, Chinese Academy of Medical Science and Peking Union Medical College, Beijing 100730, China

## Abstract

**Objective:**

Delays in skeletal maturity are related to bone mass and fracture risk in children, but the factors that determine it are unknown. We aimed to identify the association between insulin-like growth factor-1 (IGF-1) and skeletal maturation before and after growth hormone (GH) treatment.

**Methods:**

In this retrospective cohort study, we observed 783 short children and adolescents, 229 of whom received GH therapy. Skeletal maturation was assessed based on the difference between bone age (BA) and chronological age (CA) (noted as BA-CA). Anthropometric data and laboratory values were measured, and BA was evaluated using the Greulich and Pyle method.

**Results:**

The delayed BA group was defined as BA‐CA < −2 SD (*n* = 457), and the occurrence rate of BA delay was 58.37%. A nonlinear relationship was observed between the IGF-1 standard deviation score (IGF-1 SDS) and BA-CA before and after GH therapy. Before GH therapy, there was a significant positive association between the IGF-1 SDS and BA-CA when the IGF-1 level was greater than -2 SDS (*β* 0.17, 95% CI 0.08, 027; *P* < 0.001). However, we did not observe a significant relationship between the IGF-1 SDS and BA-CA when the IGF-1 level was lower than -2 SDS (*β* 0.07, 95% CI -0.12, 0.26; *P* = 0.454). After GH therapy, there was a significant positive association between the IGF-1 SDS and BA-CA when the IGF-1 level was lower than 2 SDS (*β* 0.20, 95% CI 0.12, 028; *P* < 0.001). However, we did not observe a significant relationship between the IGF-1 SDS and BA-CA when the IGF-1 level was greater than 2 SDS (*β* -0.03, 95% CI -0.33, 0.27; *P* = 0.866).

**Conclusion:**

BA is more delayed in short children and adolescents. There is a nonlinear relationship between IGF-1 and BA maturation in short children before and after GH treatment. These findings suggest that a low level of IGF-1 may contribute to BA delay in short children and adolescents.

## 1. Introduction

Bone age (BA) is an indicator of physical maturation in childhood and adolescence and is particularly helpful in the clinical workup of children with growth delays [1]. BA assessment is a routine procedure in pediatric radiology departments to identify skeletal maturation delay or advancement [2]. When BA is younger than the chronological age (CA) by more than one year, BA delay is diagnosed [3]. Delayed BA in children is associated with decreased bone mineral density and increased fracture risk [4], and BA delay is common in children with short stature and adolescents [5]. Bone maturation is a complex phenomenon influenced by several hormonal, nutritional, socioeconomic, and genetic factors [6–8]. However, the skeletal development and maturity of children and adolescents mainly depends on the growth hormone/insulin growth factor-1 (GH/IGF-1) axis. The GH/IGF-1 axis provides the main stimulus for bone growth regulation by activating the osteoblast differentiation program and stimulating chondrocyte proliferation in the growth plate [9]. In vivo models, a lack of IGF-1 led to growth retardation and decreased bone formation rates [10, 11]. A clinical study reported that type 1 diabetic children with abnormalities of the GH/IGF-1 axis fail to achieve normal peak bone mass and had an increased likelihood of developing osteoporosis and fractures later in life [12].

GH treatment can effectively increase predicted adult height in children with short stature. After GH therapy, elevated serum IGF-1 or GH levels can stimulate growth plate development and lead to BA progression. The rate of BA progression during GH therapy has an important effect on the predicted adult height; this rate varies among patients receiving GH and can be within the normal or advanced range [13–16]. A randomized controlled GH trial showed a significant acceleration in BA maturation in the GH treatment group compared to the non-GH treatment group [17]. However, a retrospective cohort study demonstrated that elevated serum IGF-1 levels were not related to BA progression [6]. There are controversies regarding the changes in IGF-1 and the development of BA after GH treatment, and data on the BA progression rate in short stature patients receiving GH treatment are insufficient. The aim of this study was to examine the association between IGF-1 and skeletal maturation before and after GH treatment in short Chinese children and adolescents.

## 2. Subjects and Methods

### 2.1. Study Population

**Figure 1:**
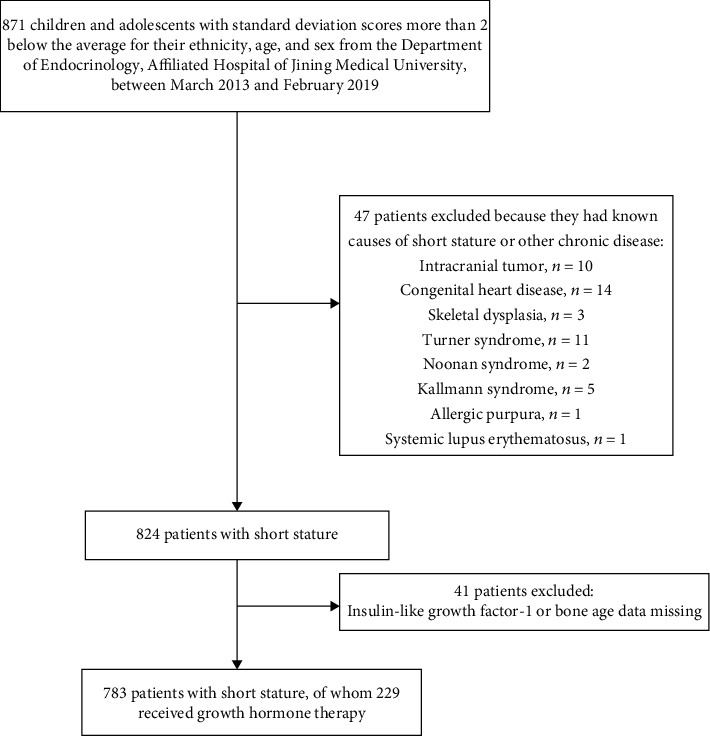
Flow chart of the study population.

The subjects were enrolled from March 2013 to February 2019 at the Department of Endocrinology, Affiliated Hospital of Jining Medical University. They are part of the Growth and Development Diseases in Shandong Province (GDDSD) cohort study (http://www.chictr.org.cn, ChiCTR1900026510). A total of 783 children and adolescents (553 males and 230 females, aged 10.2 ± 3.5 years) with short stature whose height was more than two standard deviation scores (SDS) below the median height for the relevant age and sex were enrolled. Among them, 229 subjects received GH and were followed up. The clinical characteristics of the children who did and did not receive GH were similar (Supplementary Table 1). The exclusion criteria were as follows: short stature with precocious puberty, congenital adrenal hyperplasia, cartilage dysplasia, and chromosomal or genetic abnormalities such as Turner syndrome. The flow chart of the study population is shown in Figure 1. Subjects were divided into two groups based on the difference between BA and CA (denoted as BA-CA). The delayed BA group was defined as BA‐CA < −2 SD, and the normal BA group was defined as 2 SD ≤ BA‐CA ≤ −2 SD.

Ethics approval was obtained from the Human Ethics Committee of the Affiliated Hospital of Jining Medical University (Shandong, China). All of the patients' families were informed of the aims of the study, and written informed consent forms were signed by all of the participants' parents.

### 2.2. Anthropometric Measurements

Height and weight were assessed in light clothing, with shoes removed, following standard procedures. Body height was measured to the nearest 0.1 cm with a Best Industrial Stadiometer (Nantong Best Industrial Co., Ltd., Jiangsu, China). A weighing scale capacity of 120 kg and a precision of 0.1 kg (Wuxi Weigher Factory Co., Ltd., Jiangsu, China) was used to measure body weight. Height was expressed as the SDS based on normative values for Chinese children [18]. BMI was calculated as the weight divided by the height in meters squared, and the SDS was calculated according to 2009 Chinese children and adolescent growth charts [19]. Puberty stage was evaluated by physical examination based on the Tanner stages [20]. The following criteria were considered prepubescent: boys with no pubic hair and a testicular volume less than 4 ml and girls with no pubic hair and no breast development.

### 2.3. Laboratory Measurements

The serum concentration of IGF-1 was estimated based on a chemiluminescence assay (DPC IMMULITE 1000 analyzer, SIEMENS, Germany) with intra-assay and interassay coefficients of variation of 3.0% and 6.2%, respectively. The alkaline phosphatase (ALP) level in the serum was detected by a biochemical autoanalyzer (Cobas c702, Roche; Shanghai, China). Thyroid function, including free triiodothyronine (FT3), free thyroxine (FT4), and thyrotrophic hormone (TSH), was tested by a luminescence immunoassay system (Cobas e602, Roche; Shanghai, China). The measures of the intra-assay and interassay coefficients of variation for follicle stimulating hormone (FSH) were 2.9% and 2.7%, those for luteinizing hormone (LH) were 2.6% and 3.2%, those for estradiol (E2) were 9.2% and 4.5%, and those for testosterone (T) were 8.5% and 4.2%, respectively, and they were determined with an immunoassay system (ADVIA Centaur XP, SIEMENS, Germany). The IGF-1 SDS for age and sex was calculated according to IGF-1 levels determined in Japan for the same age and sex healthy children and adolescents [21].

### 2.4. X-Ray Bone Age Assessment

BA and the BA SD were assessed using a radiograph of the left hand and wrist (Ysio SIEMENS, Germany). All radiographs were analyzed by the same independent experienced pediatric radiologist blinded to the patients' chronological ages using the Greulich and Pyle method [2]. One hundred X-rays were randomly selected for assessment by a second experienced pediatric radiologist to evaluate the interobserver variation and were reassessed by the first reader after a 2-month interval to evaluate the interobserver variation. Skeletal maturation was evaluated by the difference between BA and CA. For 100 randomly selected children who were reassessed, the mean BA for the first evaluation was 8.64 ± 3.89, and the mean BA for the second evaluation was 8.63 ± 3.89. There was no difference between the two groups (*P* > 0.05). In addition, the BA of children with and without secondary evaluations were 8.64 ± 3.89 and 8.15 ± 3.84, respectively, and there was also no difference between the two groups (*P* > 0.05).

### 2.5. Statistical Analysis

Continuous variables are displayed as the mean ± standard deviation, and categorical variables are expressed as the numbers and percentages. To compare differences between two groups, Student's *t*-test was used for normally distributed variables, the Kruskal-Wallis test was used for nonnormally distributed variables, and the chi-square test and Fisher's exact test were used for categorical variables. A univariate model was used to examine whether the IGF-1 SDS and other anthropometric and biochemical variables were associated with BA-CA. A smooth curve was fitted to explore the relationship between the IGF-1 SDS and BA-CA. Multivariate piecewise linear regression was further used to examine the threshold of the association between the IGF-1 SDS and BA-CA according to the smooth curve before GH therapy. A generalized additive mixed model was used to analyze the changes over time in IGF-1 and BA from baseline during GH therapy and to analyze the relationship between IGF-1 and skeletal maturation after GH treatment. A two-tailed *P* < 0.05 was considered statistically significant in all analyses. The statistical analysis was performed with R 3.4.3 (https://www.R-project.org) and EmpowerStats (https://www.empowerstats.com, X&Y Solutions, Inc., Boston, MA).

## 3. Results

### 3.1. Clinical Characteristics of the Included Subjects

Data on the clinical characteristics of all study participants are described in Table 1. Of the 783 subjects, 457 (58.37%) were categorized into the delayed BA group and 326 (41.63%) were categorized into the normal BA group. The mean CA and BA of the study group were 10.2 ± 3.5 years and 8.2 ± 3.8 years, respectively. The median and interquartile range of the IGF-1 SDS and BA-CA was -1.03 (-1.86–-0.19) and -1.98 (-2.84–-1.28), respectively. The BA-CA, height SDS, weight, BMI SDS, and IGF-1 SDS in the delayed BA group were significantly lower than those in the normal BA group (all *P* < 0.01).

### 3.2. Factors Associated with BA-CA in the Subjects

Table 2 shows the associations between BA-CA and all tested variables according to univariate analysis. A significant positive relationship between the IGF-1 SDS and BA-CA was observed (*P* < 0.001). Other variables, including sex, pubertal stage, height SDS, weight, BMI SDS, E2, T, LH (*P* < 0.001), and FSH (*P* = 0.006), were all positively associated with BA-CA. However, there were no significant associations between BA-CA and FT3, FT4, TSH, and ALP (all *P* > 0.05).

### 3.3. Independent Association between IGF-1 SDS and BA-CA at Baseline

**Figure 2:**
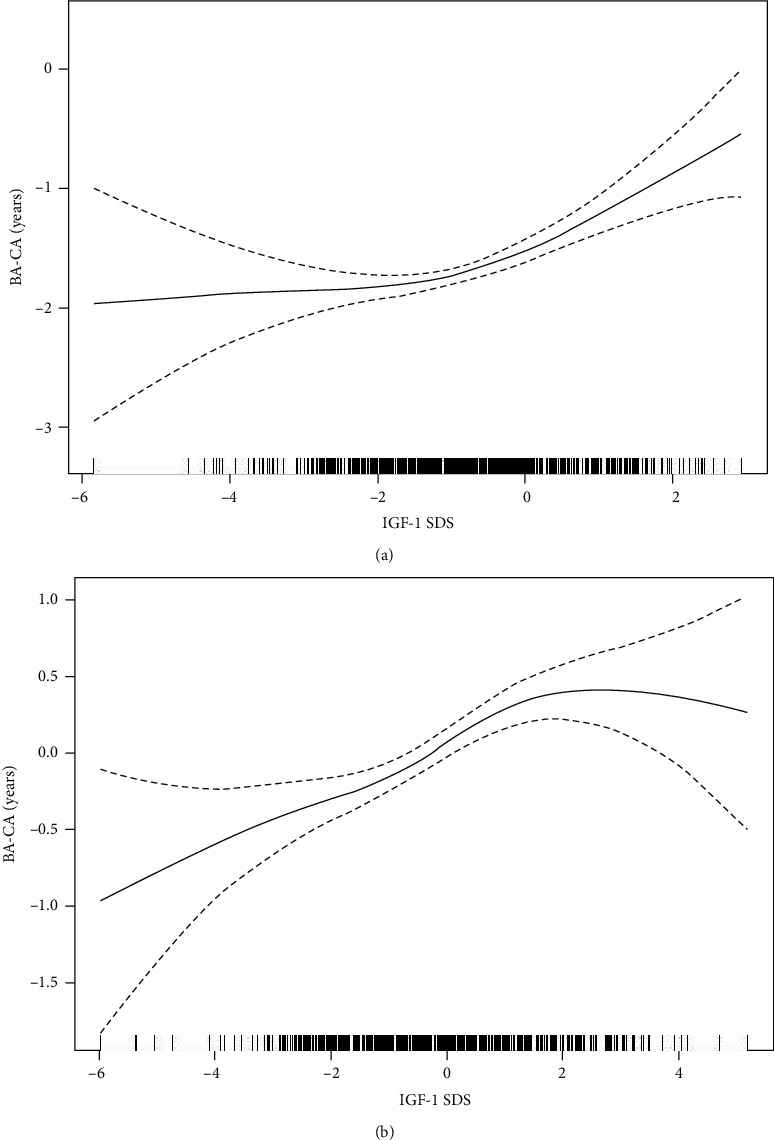
The association between the IGF-1 SDS and BA-CA before (a) (*N* = 783) and after GH therapy (b) (*N* = 229). Adjustment variables: age, sex, BMI, pubertal stage, E2, T, LH, FSH. (b) Increased GH dose and GH treatment duration (*N* = 229). IGF-1 SDS: insulin-like growth factor-1 standard deviation score; BA-CA: bone age-chronological age; GH: growth hormone; BMI: body mass index.

A smooth curve was fitted after adjustment for potential baseline confounding factors before GH treatment. A nonlinear relationship was observed between the IGF-1 SDS and BA-CA, and a two-stage change and an inflection point were observed in the resultant curve (Figure 2(a)). In addition, we further applied a multivariate piecewise regression to evaluate the independent relationship between the IGF-1 SDS and BA-CA in line with the fitted smooth curve, and the inflection point was an IGF-1 SDS of -2 (Table 3). Analysis of the threshold effects indicated that BA-CA increased with increasing IGF-1 SDS when the IGF-1 level was more than -2 SDS (*β* 0.17, 95% CI 0.08, 027; *P* < 0.001). However, we did not observe a significant relationship between the IGF-1 SDS and BA-CA when the IGF-1 level was lower than -2 SDS (*β* 0.07, 95% CI -0.12, 0.26; *P* = 0.454).

### 3.4. Generalized Additive Mixed Model

**Figure 3:**
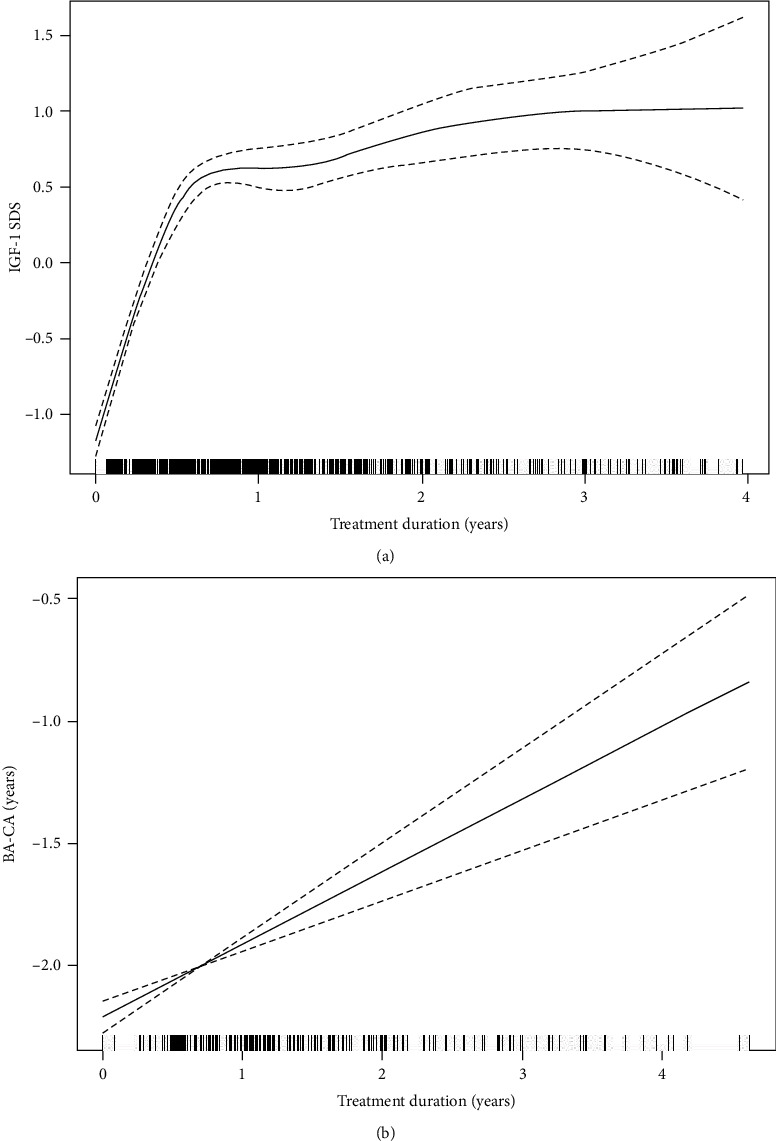
IGF-1 SDS (a) and BA-CA changes (b) during GH treatment in short children and adolescents are shown (*N* = 229). IGF-1 SDS: insulin-like growth factor-1 standard deviation score; BA: bone age; CA: chronological age; GH: growth hormone.

After adjustment for potential confounding factors, there was a nonlinear relationship between the IGF-1 SDS and BA-CA after GH treatment (Figure 2(b)). As the IGF-1 SDS level increased, the displayed BA-CA initially increased and then plateaued. As shown in Table 3, during follow-up among the children receiving GH therapy, there was a significant positive association between the IGF-1 SDS and BA-CA when the IGF-1 level was lower than 2 SDS (*β* 0.20, 95% CI 0.12, 028; *P* < 0.001). However, we did not observe a significant relationship between the IGF-1 SDS and BA-CA when the IGF-1 level was greater than 2 SDS (*β* -0.03, 95% CI -0.33, 0.27; *P* = 0.866). In addition, as shown in Figure 3, changes in the IGF-1 SDS and BA-CA during GH therapy over time were described by a generalized additive mixed model. Figures 3(a) and 3(b) depict the changes in the serum IGF-1 SDS and BA-CA at different follow-up times, respectively. There was a positive association between the level of IGF-1 SDS and the duration of therapy within one year of GH treatment (*β* 2.18; 95% CI: 1.92, 2.43; *P* < 0.001). After one year of GH treatment, there was no significant relationship between IGF-1 and the duration of treatment (*β* 0.07; 95% CI: -0.10, 0.24; *P* = 0.399). In addition, there was a positive association between BA-CA and GH treatment duration (*β* 0.30; 95% CI: 0.21, 0.39; *P* < 0.001).

## 4. Discussion

This retrospective cohort study revealed that children and adolescents with short stature were prone to BA delay (58.37%). There was a nonlinear relationship between IGF-1 and BA maturation in children before and after GH treatment. Furthermore, we revealed a threshold effect based on the BA delay, and the IGF-1 SDS turning point was -2 before GH therapy and 2 after GH therapy. The positive relationship between the IGF-1 SDS and BA-CA was significant only when the IGF-1 SDS was >-2 before GH therapy and when the IGF-1 SDS was <2 after GH therapy.

BA is a surrogate for developmental age, or physiological maturity, which represents age more truthfully than chronological age and is important for predicting adult height. A delayed BA in children is associated with decreased bone mineral density and increased fracture risk. Jones and Ma reported that skeletal maturation was associated with both bone mass and upper limb fracture risk (especially of the hand) in children aged 9-16 years, and this relationship remained after adjustment for bone density [4]. A delay in BA is often observed in children with short stature [5], and our result is in accordance with this conclusion. Our study revealed that children and adolescents with short stature were prone to BA delay and that the height SDS was positively associated with BA-CA. Our data are in agreement with the findings of a previous study [22], which reported that nonobese children and adolescents who were shorter (lower height *Z*-score) had more delayed BA. In addition, in one large cross-sectional study with 665 males and 1018 females aged 3-25 years conducted in North Sudan, investigators reported that skeletal maturity was relatively more delayed in the low height group [23]. This suggests that we should pay more attention to BA delay in short children and adolescents.

In the present study, we found that the IGF-1 SDS was positively associated with BA-CA and that lower than normal IGF-1 levels may explain why children with short stature are prone to delayed BA. A previous study illustrated a strong association between IGF-1 and skeletal acquisition, demonstrating that during childhood, other hormones, notably IGF-1, may play a more prominent role in bone mineral accrual than vitamin D [24]. It is well established that IGF-1 signaling is essential for osteoblast differentiation [25]. The GH/IGF-1 axis stimulates chondrocyte proliferation on the growth plate by activating the osteoblast differentiation program, regulates phosphate reabsorption of renal tubules and the activation of 25 hydroxyvitamin D31a hydroxylase, and provides the main stimulation for bone growth regulation [9]. Reinehr et al. [26] demonstrated that the IGF-1 concentrations were positively associated with skeletal maturation in 356 obese children aged 4-15 years. However, they conducted the study in a relatively small sample of obese children, and a positive association was only reported with Spearman's rank correlation. We conducted our study with a relatively large sample of short children and further explored the independent effect of IGF-1 on BA delay through multivariate regression. Interestingly, we found a nonlinear relationship between the IGF-1 SDS and BA delay in short children and adolescents. IGF-1 can promote bone maturation and is positively associated with BA-CA only when the level of the IGF-1 SDS is greater than -2. However, when the level of the IGF-1 SDS is less than -2, the relationship between the IGF-1 SDS and the BA-CA is not significant, possibly because other factors are influencing the BA delay. We believe that the explanation for the nonlinear relationship between the IGF-1 SDS and BA delay and the existence of a threshold (-2) in our study was that the range in the level of IGF-1 is broad; this is especially true for the low level of IGF-1 compared with the level in a previous study [26] conducted in obese children with the normal-high serum IGF-1 levels. Our findings suggest that IGF-1 may play a well-known role in skeletal maturation, which is consistent with a previous study [26]. Children with short stature, especially those with growth hormone deficiency (GHD), should receive GH therapy to increase height growth and improve BA delay. However, not all children with delayed BA require GH therapy. For example, children with constitutional delay of growth and puberty (CDGP), which refers to a diagnosis that can be definitively made only retrospectively, enter puberty later than the norm, and this condition has been completely attributed to a delay in androgen function. Androgen treatment is often used in adolescents with CDGP to improve delayed bone maturation [27].

Previous studies have also reported an increase in serum IGF-1 levels after long-term GH treatment, but the average IGF-1 SDS level remained within the range of 2 SD [28–30], and our finding is consistent with this. This finding might be related to the slow release of GH, which promotes the production of IGF-1 in the liver. The IGF-1 SDS sharply increased during the first year of GH treatment, and this observation was also made in a previous study [6]. In our study, after adjusting for potential confounders, including BMI, this relationship remained. BMI is known to be a regulator of the GH/IGF-1 axis [31]. Previous studies have shown that there is a relationship between BMI and IGF-1 in children and adults [32, 33]. The reason may be that BMI is related to GH, and it is known that obesity is associated with impaired GH secretion [34].

Interestingly, we further explored the relationship between IGF-1 and BA maturation after GH treatment and observed that there is a nonlinear relationship between the IGF-1 SDS and BA-CA. The results of this study suggest that when the IGF-1 levels are less than 2 SD, BA matures with increasing IGF-1 levels, but no association was found between BA and IGF-1 levels when the IGF-1 level was greater than 2 SD. This is also consistent with the level of IGF-1 that guarantees safety in GH therapy [35].

There are some limitations of this study. First, only those children receiving GH treatment were followed in this study, and we were unable to analyze changes in the IGF-1 SDS over time in children not receiving GH therapy. Second, we were unable to assess multiple factors associated with the regulation of skeletal maturation, such as leptin and 25-hydroxyvitamin D, and further studies are needed to explore the effects of IGF-1 on bone formation markers. Finally, the present findings are only applicable to children with short stature, and different results might be observed in other groups.

In conclusion, we observed that the rate of occurrence of BA delay in short Chinese children and adolescents was 58.37%. IGF-1 levels and BA maturation increase with GH treatment in short children and adolescents, and both are within the clinically acceptable range. IGF-1 is related to BA maturation to a certain extent before and after GH treatment. These findings suggest that a low level of IGF-1 may contribute to BA delay in short children and adolescents, and additional prospective studies are needed to further investigate the underlying mechanisms of the relationship between skeletal maturation and short stature.

## Figures and Tables

**Table 1:** Clinical and laboratory characteristics of the subjects.

Variable	Total	Delayed bone age	Normal bone age	*P*
Number (%)	783 (100%)	457 (58.37%)	326 (41.63%)	—
GH treatment (%)	229 (100%)	135 (58.95%)	94 (41.05%)	—
Sex (male %)	553 (70.63%)	342 (74.84%)	211 (64.72%)	0.002
Chronological age (years)	10.2 ± 3.5	9.3 ± 3.5	11.5 ± 3.0	<0.001
Bone age (years)	8.2 ± 3.8	6.5 ± 3.4	10.6 ± 3.0	<0.001
BA-CA (years)	-1.98 (-2.84–-1.28)	-2.70 (-3.32–-2.22)	-1.09 (-1.43–-0.59)	<0.001
Height (cm)	125.79 ± 18.07	120.49 ± 17.70	133.23 ± 15.86	<0.001
Height SDS	−2.66 ± 0.59	−2.71 ± 0.63	−2.60 ± 0.52	0.007
Body weight (kg)	27.72 ± 10.85	24.37 ± 9.40	32.42 ± 11.01	<0.001
BMI (kg/m^2^)	16.80 ± 2.84	16.16 ± 2.40	17.70 ± 3.15	<0.001
BMI SDS	−0.30 ± 1.12	−0.39 ± 1.08	−0.16 ± 1.16	0.005
IGF-1 (ng/ml)	164.00 (92.30-253.50)	128.50 (76.62-196.00)	232.00 (139.00-340.00)	<0.001
IGF-1 SDS	-1.03 (-1.86–-0.19)	-1.21 (-1.93–-0.52)	-0.58 (-1.63-0.35)	<0.001
FT3 (pmol/l)	6.42 ± 1.20	6.38 ± 1.05	6.47 ± 1.38	0.306
FT4 (pmol/l)	19.57 ± 4.19	17.72 ± 2.72	22.16 ± 5.84	0.261
TSH (mIU/l)	2.96 ± 1.38	3.01 ± 1.37	2.88 ± 1.38	0.178
ALP (U/l)	317.08 ± 138.79	301.08 ± 134.95	338.98 ± 141.22	<0.001
E2 (pg/ml)	18.15 (11.80-26.31)	16.64 (11.80-23.41)	20.80 (13.48-28.66)	<0.001
T (pg/ml)	0.23 (0.12-0.47)	0.20 (0.10-0.32)	0.29 (0.16-0.97)	<0.001
FSH (mIU/ml)	2.34 (1.01-3.96)	1.75 (0.73-3.43)	3.14 (1.78-4.68)	0.011
LH (mIU/ml)	0.22 (0.10-1.40)	0.10 (0.04-0.94)	0.82 (0.10-1.99)	<0.001
Pubertal stage				<0.001
In prepuberty (%)	579 (73.95%)	385 (84.25%)	194 (59.51%)	
In puberty (%)	204 (26.05%)	72 (15.75%)	132 (40.49%)	

Abbreviations: GH: growth hormone; BA-CA: bone age-chronological age; height SDS: height standard deviation scores; BMI: body mass index; BMI SDS: body mass index standard deviation scores; IGF-1: insulin-like growth factor-1; IGF-1 SDS: insulin-like growth factor-1 standard deviation scores; FT3: triiodothyronine; FT4: free thyroxine; TSH: thyrotrophic hormone; ALP: alkaline phosphatase; E2: estradiol; T: testosterone; FSH: follicle stimulating hormone; LH: luteinizing hormone. Delayed BA group was defined as BA‐CA < −2 SD, and normal BA group was defined as 2 SD ≤ BA‐CA ≤ −2 SD. Normal distribution of data was presented as mean ± standard deviation; nonnormal distribution of data was presented as median (interquartile range) and categorical data using number (percentage). *P* < 0.05 is considered to be statistically significant.

**Table 2:** Associations between BA-CA and different variables.

Variables	*β*	(95% CI)	*P*
Height (cm)	0.01	(0.01, 0.01)	<0.001
Height SDS	0.44	(0.30, 0.58)	<0.001
Body weight (kg)	0.03	(0.02, 0.03)	<0.001
BMI (kg/m^2^)	0.11	(0.08, 0.14)	<0.001
BMI SDS	0.25	(0.17, 0.32)	<0.001
IGF-1 (ng/ml)	0.01	(0.01, 0.02)	<0.001
IGF-1 SDS	0.22	(0.15, 0.28)	<0.001
FT3 (pmol/l)	0.07	(0.00, 0.14)	0.050
FT4 (pmol/l)	0.01	(-0.01, 0.01)	0.383
TSH (mIU/l)	-0.01	(-0.07, 0.05)	0.757
ALP (U/l)	0.01	(-0.01, 0.01)	0.135
E2 (pg/ml)	0.01	(0.01, 0.02)	<0.001
T (pg/ml)	0.24	(0.15, 0.33)	<0.001
FSH (mIU/ml)	0.02	0.02 (0.01, 0.04)	0.006
LH (mIU/ml)	0.15	(0.09, 0.21)	<0.001
Sex			
Male	Reference		
Female	0.41	(0.23, 0.59)	<0.001
Pubertal stage			
In prepuberty (%)	Reference		
In puberty (%)	0.49	(0.31, 0.68)	<0.001

Abbreviations: BA-CA: bone age-chronological age; height SDS: height standard deviation scores; BMI: body mass index; BMI SDS: body mass index standard deviation scores; IGF-1: insulin-like growth factor-1; IGF-1 SDS: insulin-like growth factor-1 standard deviation scores; FT3: triiodothyronine; FT4: free thyroxine; TSH: thyrotrophic hormone; ALP: alkaline phosphatase; E2: estradiol; T: testosterone; FSH: follicle stimulating hormone; LH: luteinizing hormone. *P* < 0.05 is considered to be statistically significant.

**Table 3:** Threshold effect analysis of the association between the IGF-1 SDS and BA-CA before and after GH therapy by multivariate piecewise regression.

Inflection point of IGF-1 SDS	BA-CA	
	*β* (95% CI)	*P*
Baseline (*N* = 783)		
>-2	0.17 (0.08, 0.27)	<0.001
≤-2	0.07 (-0.12, 0.26)	0.454
Follow-up (*N* = 229)		
<2	0.20 (0.12, 0.28)	<0.001
≥2	-0.03 (-0.33, 0.27)	0.866

Adjustment variables: age, sex, pubertal stage, height SDS, weight, BMI SDS, E2, T, LH, and FSH. Height SDS: height standard deviation scores; BMI SDS: body mass index standard deviation scores; E2: estradiol; T: testosterone; FSH: follicle stimulating hormone; LH: luteinizing hormone. *P* < 0.05 is considered to be statistically significant.

## Data Availability

The data used to support the findings of this study are available from the corresponding author upon request.

## Supplementary Materials

Supplementary Materials Supplementary Table 1: clinical characteristics of the subjects with and without GH therapy.

## References

[1] Martin D. D., Wit J. M., Hochberg Z.’e. (2011). The use of bone age in clinical practice–part 1. *Hormone Research in Paediatrics*.

[2] Satoh M. (2015). Bone age: assessment methods and clinical applications. *Clinical Pediatric Endocrinology*.

[3] Subspecialty Group of Endocrinologic (2008). Guidelines for diagnosis and treatment of children with short stature. *Chinese Journal of Pediatrics*.

[4] Jones G., Ma D. (2005). Skeletal age deviation assessed by the Tanner-Whitehouse 2 method is associated with bone mass and fracture risk in children. *Bone*.

[5] Su W., Wang S., Zhu Z. (2015). Etiology and bone age of 2132 children with short stature. *Journal of Clinical Pediatrics*.

[6] Kang M. J., Kim E. Y., Shim Y. S. (2019). Factors affecting bone age maturation during 3 years of growth hormone treatment in patients with idiopathic growth hormone deficiency and idiopathic short stature. *Medicine*.

[7] Cole T. J., Rousham E. K., Hawley N. L., Cameron N., Norris S. A., Pettifor J. M. (2015). Ethnic and sex differences in skeletal maturation among the Birth to Twenty cohort in South Africa. *Archives of Disease in Childhood*.

[8] Vandewalle S., Taes Y., Fiers T. (2014). Sex steroids in relation to sexual and skeletal maturation in obese male adolescents. *The Journal of Clinical Endocrinology & Metabolism*.

[9] Giustina A., Mazziotti G., Canalis E. (2008). Growth hormone, insulin-like growth factors, and the skeleton. *Endocrine Reviews*.

[10] McMichael B. K., Jeong Y. H., Auerbach J. A. (2017). The RhoGAP Myo9b promotes bone growth by mediating osteoblastic responsiveness to IGF-1. *Journal of Bone and Mineral Research*.

[11] Chen C. Y., Tseng K. Y., Lai Y. L., Chen Y. S., Lin F. H., Lin S. (2017). Overexpression of insulin-like growth factor 1 enhanced the osteogenic capability of aging bone marrow mesenchymal stem cells. *Theranostics*.

[12] Raisingani M., Preneet B., Kohn B., Yakar S. (2017). Skeletal growth and bone mineral acquisition in type 1 diabetic children; abnormalities of the GH/IGF-1 axis. *Growth Hormone & IGF Research*.

[13] Zadik Z., Chalew S., Zung A., Landau H., Kowarski A. A. (1994). Effect of long-term growth hormone therapy on bone age and pubertal maturation in boys with and without classic growth hormone deficiency. *The Journal of Pediatrics*.

[14] Frindik J. P., Kemp S. F., Sy J. P. (1999). Effects of recombinant human growth hormone on height and skeletal maturation in growth hormone-deficient children with and without severe pretreatment bone age delay. *Hormone Research in Paediatrics*.

[15] Kawai M., Momoi T., Yorifuji T., Yamanaka C., Sasaki H., Furusho K. (1997). Unfavorable effects of growth hormone therapy on the final height of boys with short stature not caused by growth hormone deficiency. *The Journal of Pediatrics*.

[16] Hopwood N. J., Hintz R. L., Gertner J. M. (1993). Growth response of children with non-growth-hormone deficiency and marked short stature during three years of growth hormone therapy. *The Journal of Pediatrics*.

[17] Arends N. J. T., Boonstra V. H., Mulder P. G. H. (2003). GH treatment and its effect on bone mineral density, bone maturation and growth in short children born small for gestational age: 3-year results of a randomized, controlled GH trial. *Clinical Endocrinology*.

[18] Li H., Ji C. Y., Zong X. N., Zhang Y. Q. (2009). Height and weight standardized growth charts for Chinese children and adolescents aged 0 to 18 years. *Chinese Journal Of Pediatrics*.

[19] Li H., Ji C. Y., Zong X. N., Zhang Y. Q. (2009). Body mass index growth curves for Chinese children and adolescents aged 0 to 18 years. *Chinese Journal Of Pediatrics*.

[20] Wright C. M., Ahmed L., Dunger D. B., Preece M. A., Cole T. J., Butler G. (2012). Can we characterise growth in puberty more accurately? Validation of a new puberty phase specific (PPS) growth chart. *Archives of Disease in Childhood*.

[21] Isojima T., Shimatsu A., Yokoya S. (2012). Standardized centile curves and reference intervals of serum insulin-like growth factor-I (IGF-I) levels in a normal Japanese population using the LMS method. *Endocrine Journal*.

[22] McCormack S. E., Chesi A., Mitchell J. A. (2017). Relative skeletal maturation and population ancestry in non-obese children and adolescents. *Journal of Bone and Mineral Research*.

[23] Elamin F., Abdelazeem N., Elamin A., Saif D., Liversidge H. M. (2017). Skeletal maturity of the hand in an East African group from Sudan. *American Journal of Physical Anthropology*.

[24] Breen M. E., Laing E. M., Hall D. B. (2011). 25-Hydroxyvitamin D, insulin-like growth factor-I, and bone mineral accrual during growth. *The Journal of Clinical Endocrinology & Metabolism*.

[25] Crane J. L., Zhao L., Frye J. S., Xian L., Qiu T., Cao X. (2013). IGF-1 signaling is essential for differentiation of mesenchymal stem cells for peak bone mass. *Bone Research*.

[26] Reinehr T., de Sousa G., Wabitsch M. (2006). Relationships of IGF-I and androgens to skeletal maturation in obese children and adolescents. *Journal of Pediatric Endocrinology and Metabolism*.

[27] Lampit M., Hochberg Z. E. (2003). Androgen therapy in constitutional delay of growth. *Hormone Research in Paediatrics*.

[28] Strasburger C. J., Vanuga P., Payer J. (2017). MOD-4023, a long-acting carboxy-terminal peptide-modified human growth hormone: results of a phase 2 study in growth hormone-deficient adults. *European Journal of Endocrinology*.

[29] Fisher D. M., Rosenfeld R. G., Jaron-Mendelson M., Amitzi L., Koren R., Hart G. (2017). Pharmacokinetic and pharmacodynamic modeling of MOD-4023, a long-acting human growth hormone, in growth hormone deficiency children. *Hormone Research in Paediatrics*.

[30] Battelino T., Rasmussen M. H. J., De Schepper J. (2017). Somapacitan, a once-weekly reversible albumin-binding GH derivative, in children with GH deficiency: a randomized dose-escalation trial. *Clinical Endocrinology*.

[31] Wudy S. A., Hagemann S., Dempfle A. (2005). Children with idiopathic short stature are poor eaters and have decreased body mass index. *Pediatrics*.

[32] Yamamoto H., Kato Y. (1993). Relationship between plasma insulin-like growth factor I (IGF-I) levels and body mass index (BMI) in adults. *Endocrine Journal*.

[33] Pınar C., Firdevs B., Fatmahan A. (2013). Growth hormone/insulin-like growth factor-1? Axis as related to body mass index in patients with idiopathic short stature. *Journal of Clinical Research in Pediatric Endocrinology*.

[34] Lee H. S., Jin S. H. (2010). Influence of body mass index on growth hormone responses to classic provocative tests in children with short stature. *Neuroendocrinology*.

[35] Liu H., Wang L., Chen L. (2019). Evaluation of safety and efficacy of growth hormone therapy by IGF-1 *Z* score in children with short stature. *Advances in therapy*.

