# Supplementary material for: Association between Insulin-Like Growth Factor-1 and Relative Skeletal Maturation: A Retrospective Cohort Study of Short Children and Adolescents
Source: Biomed Res Int. 2020 Aug 13;2020:8052143. doi: 10.1155/2020/8052143 (PMC7443236; doi:10.1155/2020/8052143)
Supplement: Supplementary Materials — Supplementary Table 1: clinical characteristics of the subjects with and without GH therapy. [file 8052143.f1.doc]

**Supplementary T_ABLE_ 1: Clinical characteristics of the subjects with and without GH therapy**

| Variable | Total | With GH therapy | Without GH therapy | P |
| --- | --- | --- | --- | --- |
| Number | 783 | 229 | 554 | - |
| Sex (male %) | 553 (70.63%) | 162 (70.74%) | 391 (70.58%) | 0.989 |
| Chronological age (years) | 10.2 ± 3.5 | 10.0 ± 3.3 | 10.3 ± 3.5 | 0.257 |
| Bone age (years) | 8.2 ± 3.8 | 7.9 ± 3.6 | 8.3 ± 3.8 | 0.265 |
| Δ BA-CA (years) | -1.98 (-2.84--1.28) | -1.98 (-2.84--1.28) | -1.98 (-2.84--1.27) | 0.813 |
| Height (cm) | 125.79 ± 18.07 | 124.64 ± 17.43 | 126.40 ± 18.35 | 0.213 |
| Height SDS | -2.66 ± 0.59 | -2.66 ± 0.57 | -2.67 ± 0.60 | 0.883 |
| Body weight (kg) | 27.72 ± 10.85 | 27.58 ± 11.12 | 27.86 ± 10.78 | 0.745 |
| BMI (kg/m^2^) | 16.80 ± 2.84 | 16.99 ± 2.99 | 16.73 ± 2.77 | 0.252 |
| IGF-1 (ng/ml) | 164.00 (92.30-253.50) | 164.00 (91.90-241.00) | 166.50 (93.82-257.00) | 0.529 |
| IGF-1 SDS | -1.03 (-1.86--0.19) | -1.03 (-1.86--0.19) | -1.05 (-1.87--0.19) | 0.633 |
| FT3（pmol/L） | 6.42 ± 1.20 | 6.44 ± 1.21 | 6.40 ± 1.19 | 0.711 |
| FT4（pmol/L） | 19.57 ± 4.19 | 17.49 ± 2.97 | 20.41 ± 6.20 | 0.492 |
| TSH（mIU/L） | 2.96 ± 1.38 | 2.92 ± 1.26 | 2.97 ± 1.39 | 0.667 |
| ALP (U/L) | 317.08 ± 138.79 | 329.85 ± 132.71 | 310.24 ± 141.06 | 0.098 |
| E2 (pg/ml) | 18.15 (11.80-26.31) | 17.23 (11.80-24.72) | 18.37 (12.06-26.68) | 0.066 |
| T (pg/ml) | 0.23 (0.12-0.47) | 0.21 (0.12-0.40) | 0.24 (0.12-0.49) | 0.225 |
| FSH (mIU/ml) | 2.34 (1.01-3.96) | 2.19 (0.92-4.08) | 2.41 (1.06-3.91) | 0.488 |
| LH (mIU/ml) | 0.22 (0.10-1.40) | 0.19 (0.10-1.39) | 0.23 (0.10-1.47) | 0.808 |
| Pubertal stage |  |  |  | 0.512 |
| In prepuberty (%) | 579 (73.95%) | 173 (75.55%) | 406 (73.29%) |  |
| In puberty (%) | 204 (26.05%) | 56 (24.45%) | 148 (26.71%) |  |

Abbreviations: GH: growth hormone; Δ BA-CA: bone age-chronological age; height SDS: height standard deviation scores; BMI: body mass index; IGF-1 SDS: insulin like growth factor-1 standard deviation score; ALP: alkaline phosphatase; FT3: triiodothyronine; FT4: free thyroxine; TSH: thyrotrophic hormone; E2: estradiol; T: testosterone; FSH: follicle stimulating hormone; LH: luteinizing hormone. Continuous variables are expressed as the means ± standard deviations. Nonnormally distributed data are presented as the medians (interquartile ranges), and categorical data are presented as the numbers (percentages). P < 0.05 was considered to be statistically significant.
